# Variable Clinical Features in a Large Family With Diamond Blackfan Anemia Caused by a Pathogenic Missense Mutation in *RPS19*

**DOI:** 10.3389/fgene.2022.914141

**Published:** 2022-07-18

**Authors:** Sarah Cole, Neelam Giri, Blanche P. Alter, D. Matthew Gianferante

**Affiliations:** ^1^ Clinical Genetics Branch, Division of Cancer Epidemiology and Genetics, National Cancer Institute, Bethesda, MD, United States; ^2^ Walter Reed National Military Medical Center, Bethesda, MD, United States

**Keywords:** DBA, genotype, phenotype, ribosomopathy, inherited bone marrow failure syndrome

## Abstract

**Introduction:** Diamond Blackfan anemia (DBA) is an autosomal dominant ribosomopathy caused predominantly by pathogenic germline variants in ribosomal protein genes. It is characterized by failure of red blood cell production, and common features include congenital malformations and cancer predisposition. Mainstays of treatment are corticosteroids, red blood cell transfusions, and hematologic stem cell transplantation (HSCT). Despite a better understanding of the genotype of DBA, the biological mechanism resulting in the clinical phenotype remains poorly understood, and wide heterogeneity can be seen even within a single family as depicted here.

**Case Description:** Thirty family members enrolled in the National Cancer Institute inherited bone marrow failure syndromes study were evaluated with detailed medical questionnaires and physical examinations, including 22 in the family bloodline and eight unrelated partners. Eight participants had been previously told they had DBA by clinical criteria. Targeted germline *RPS19* testing was done on all family members. A pathogenic heterozygous missense mutation in *RPS19* (p.R62Q, c.185G > A) was detected in ten family members, including one person previously presumed unaffected. Eight family members presented with macrocytic anemia in infancy; all of whom were responsive to prednisone. Four family members became treatment independent; however, one individual became transfusion-dependent 36 years later following an episode of pneumonia. One prednisone responsive individual electively discontinued steroid treatment, and lives with severe anemia. One prednisone responsive individual died at age 28 from a stroke. Two family members developed colorectal cancer in their fifties; one had never required treatment for anemia. None had major congenital anomalies.

**Discussion:** This large family with DBA demonstrates the heterogeneity of phenotypes that can be seen within the same genotype. Most family members presented with steroid-responsive anemia in infancy and subtle congenital malformations, findings consistent with recent genotype-phenotype studies of *RPS* DBA. However, two family members were relatively unaffected, underscoring the importance of further studies to assess modifier genes, and epigenetic and/or environmental factors which may result in normal erythropoiesis despite underlying ribosome dysfunction. This large, multigenerational family highlights the need for individualized treatment, the importance of early cancer surveillance even in individuals with clinically mild phenotypes, and the benefit of long-term follow-up to identify late complications.

## Introduction

Diamond Blackfan anemia (DBA) is predominantly an autosomal dominant inherited red cell aplasia caused by deleterious mutations in ribosomal genes. To date, over 200 mutations in one of 26 ribosomal proteins (RP) affecting the large (RPL) or small (RPS) subunit have been identified as well as two X-linked genes, *TSR2* and *GATA1*, which encode a ribosome chaperone and a hematopoietic transcription factor targeted by altered ribosome levels (Ulirsch et al., 2018). Ribosomal protein S19 (*RPS19*) is the most commonly affected gene and accounts for approximately 25% of all DBA cases (Campagnoli et al., 2008).

As a prototypical ribosomopathy, DBA demonstrates an incongruous transition from early symptoms due to cellular hypo-proliferation to cellular hyper-proliferation with an elevated cancer risk later in life (Kampen et al., 2020). The damaging phenotype of ribosomal protein deficiency in DBA is mediated by upregulation of both p53-dependent pathways and p53-independent pathways (mRNA translation reprogramming) as a result of impaired ribosome biosynthesis (Kang et al., 2021). It is hypothesized that activation of p53 leads to apoptosis of erythroid progenitors while cellular hyperproliferation is due to altered translational capacity and genomic instability, which facilitates the production of oncogenic pathways (Dutt et al., 2011; Kang et al., 2021).

DBA is characterized by failure of red blood cell production, congenital abnormalities, poor linear growth, and cancer predisposition (Diamond et al., 1976; Vlachos et al., 2008). However, many patients may have additional symptoms or no symptoms at all. Common laboratory features of this syndrome include macrocytic anemia at less than 1 year of age, reticulocytopenia, elevated fetal hemoglobin (HbF) due to stress erythropoiesis, elevated erythrocyte adenosine deaminase (eADA), and a paucity of erythrocyte precursors in the bone marrow. Mainstays of treatment range from corticosteroids to chronic packed red blood cell (pRBC) transfusions and HSCT. About 20% of patients become treatment independent (Vlachos and Muir, 2010).

Despite a better understanding of the underlying germline genetic and ribosomal etiology of DBA, the biological mechanism resulting in variable penetrance, wide spectrum of phenotypes, and whether individuals with clinically mild phenotypes carry a life-long cancer predisposition, is still unclear. Genotype-phenotype correlations have been studied but are difficult due to the rarity of DBA, heterogenous genotype, and need for long term follow-up for many of the outcomes of interest such as cancer (Arbiv et al., 2018; Ulirsch et al., 2018; Gianferante et al., 2021). Here, we report the clinical findings of a multigenerational family with DBA caused by a missense mutation in *RPS19*. The clinical presentation of some family members was previously reported over 30 years years ago (Hunter and Hakami, 1972; Viskochil et al., 1990). We now have important genetic and clinical follow-up that spans 20 years for this family as part of the Inherited Bone Marrow Failure Syndromes Study (IBMFS) at the National Cancer Institute (NCI; www.marrowfailure.cancer.gov).

## Case Description

**FIGURE 1:**
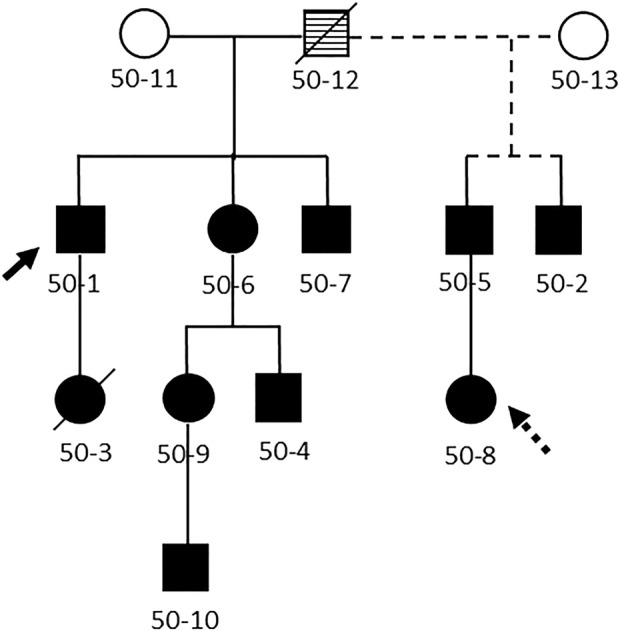
Pedigree of the family. Legend: Abbreviated pedigree, showing only those with Diamond Blackfan anemia. Solid line, first spouse; dashed line, second spouse. Filled symbol, clinically affected with DBA, open symbol, no clinical evidence for DBA, striped symbol obligate carrier. Diagonal line indicates family member is deceased. Solid arrow, proband. Dashed arrow, heterozygous mutation in *RPS19* without clinical or laboratory abnormalities.

All DBA cases (*n* = 10; Figure 1) and their unaffected relatives (*n* = 20) are participants in the IBMFS study at the NCI, an ongoing natural history and retrospective/prospective longitudinal cohort study approved by the NCI Institutional Review Board (ClinicalTrials.gov Identifier: NCT00027274) (Alter et al., 2010; Alter et al., 2018). Each participant completed comprehensive family history and medical history questionnaires. We also conducted a detailed medical record review, and four cases were evaluated at the NIH with physical examinations and laboratory studies. The laboratory studies included complete blood counts, HbF, eADA, and targeted germline DNA testing of *RPS19* since the familial missense mutation (p.R62Q, c.185G > A) was previously identified. The family initially enrolled in the study in 2003 and the most recent follow-up data are from 2022. The age at last follow-up was obtained for all subjects with the date of death serving as the age at last follow-up for those who were deceased.

A pathogenic heterozygous missense mutation in *RPS19* (p.R62Q, c.185G > A) was detected in ten family members, including one individual who was entirely asymptomatic and previously presumed unaffected (50-8; Table 1). Family member 50-8 had a normal eADA, mean corpuscular volume (MCV) and HbF. Overall, eight family members with DBA had an elevated eADA and MCV, six of whom had an elevated HbF (laboratory data were not available on patient 50-10).

**TABLE 1:** Clinical features of ten family members with Diamond Blackfan anemia due to a pathogenic heterozygous missense mutation in RPS19. Legend: Clinical information is based on medical history questionanires and detailed medical record review on all cases. Cases 50-1, 50-2, 50-3, and 50-4 were also examined at NIH by clinicians from our study. For lab values, up arrow (↑) indicates elevated and N is normal. An eADA = >0.96 EU/gm Hb was considered elevated, elevated MCV and HbF was based on >2 SD from age and sex adjusted mean, and X indicates no available labs. Abbreviations: M=male; F=female; wks=weeks of age; yrs=yrs of age; BCC=basal cell carcinoma; CRC=colorectal cancer; pRBC=packed red blood cells; NIH=National Institutes of Health; MCV=mean corpuscular volume; eADA=erythrocyte adenosine deaminase; HbF=fetal hemoglobin; L=left; b/l=bilateral. ^1^Last medical record available was at age 11.

Patient ID	Sex	Hematologic Symptoms	Hematologic Related Treatments	Lab Values			Congenital Abnormalities	Vital and Malignancy Status
				MCV	eADA	HbF		
50–1	M	Anemia at 3 weeks	Prednisone until 8 years; treatment independent from age 8 to present	↑	↑	↑	Short stature, L hypoplastic thenar	Alive at age 62; developed BCC at 44 years, CRC at 61 years
50-2	M	Anemia at birth	Prednisone treatment until 12 years; treatment independent from 12 to 47 years; steroid resistant from age 47 until present; currently pRBC transfusion-dependent and treated with Eltrombopag as per NIH treatment study (NCT04269889)	↑	↑	↑	Short stature, b/l hypoplastic thenars	Alive at age 51
50-3	F	Anemia at birth	Prednisone until time of her death	↑	↑	↑	Short stature, b/l hypoplastic thenars, hypertelorism, developmental delay, congenital heart block	Died at age 28 from a stroke
50-4	M	Anemia at birth	Prednisone responsive; electively discontinued prednisone; severely anemic and receives infrequent transfusions	↑	↑	↑	Short stature, b/l hypoplastic thenars, spina bifida occulta	Alive at age 35
50-5	M	Anemia at birth	Prednisone until 9 years; treatment independent from age 9 until present	↑	↑	N	None	Alive at age 52
50-6	F	Anemia during pregnancy at 23 years	None	↑	↑	N	B/l hypoplastic thenars	Alive at age 57; developed CRC at 56 years
50-7	M	Anemia at 6 weeks	Prednisone and requires chronic pRBC transfusion every 3 weeks	↑	↑	↑	Short stature	Alive at age 55
50-8	F	None	None^1^	N	N	N	B/l hypoplastic thenars	Alive at age 29
50-9	F	Anemia at 6 weeks	Prednisone until 12 years; treatment independent from age 12 until present	↑	↑	↑	Short stature, L hypoplastic thenar	Alive at age 30
50-10	M	Anemia at birth	Prednisone	X	X	X	None	Alive at age 5

Eight family members presented with macrocytic anemia in infancy, all of whom were responsive to prednisone. Four family members became treatment independent; however, one individual (50-2) became transfusion-dependent 36 years later following an episode of pneumonia. His treatment complications include severe glaucoma and iron overload. He is enrolled in the NIH clinical trial “Treatment of Refractory DBA with Eltrombopag” (NCT04269889) (Fan et al., 2020a; Fan et al., 2020b), a trial studying the safety and efficacy of Eltrombopag in steroid refractory DBA patients. This study is still in the early phase, and no information on therapeutic outcomes is available. One prednisone responsive individual elected to discontinue steroid treatment, undergoes infrequent pRBC transfusions, and lives with severe anemia (50-4). Another family member on prednisone died at 28 years from a stroke (50-3). One individual developed partial resistance to corticosteroids and requires pRBC transfusions every 3 weeks in addition to corticosteroids (50-7). His treatment complications include osteoporosis, pathologic fractures, and moderate iron overload. Due to his chronic conditions, he is unable to work and has been on disability since the age of 54. He was started on corticosteroids at 3 months of age, though his cumulative dose is unknown.

None of the individuals in this family with DBA had significant cardiac, craniofacial, genitourinary or limb anomalies. Seven individuals with DBA had hypoplastic thenar muscles. Six individuals had short stature, defined by < 10th percentile for height based on age and sex, in comparison to only one individual in this family without DBA. One individual was born with spina bifid occulta (50-4) and another family member had mild dysmorphic features, developmental delays and congenital heart block (50-3).

Two family members developed colorectal cancer (CRC) at 56 and 61 years of age, one of whom (50-6) had never required treatment for anemia and was only mildly anemic during pregnancy. She underwent surgical resection of her CRC, has completed radiation therapy, and is nearing completion of chemotherapy which she tolerated well without increased blood cell count suppression. The proband (50-1) had been treatment-independent for over 50 years. He underwent a colonoscopy upon CRC diagnosis in his sibling (50-6) and was identified to have localized CRC at 61 years of age which was resected. He tolerated his chemotherapy well and only required one pRBC transfusion when his hemoglobin dropped to 7.9 g/dL after his eighth and last cycle of chemotherapy. Currently he is 5 months off treatment for his CRC and he remains treatment independent from a DBA standpoint.

## Discussion

As a classic ribosomopathy, DBA illustrates how intricate cellular complexities involved in ribosome biogenesis can result in a spectrum of phenotypic abnormalities in patients with DBA. However, the factors that modulate individual responses to ribosome dysfunction remain unclear. This large, multigenerational family demonstrates the spectrum of variable penetrance and expressivity that can be seen within the same DBA genotype as well as the importance of new targeted therapeutics in this heterogeneous disease. This family additionally provides insight into cancer predisposition even for those with mild phenotypes and the importance of long-term follow-up to identify late complications in DBA.

DBA is heterogenous from both a genotype and phenotype perspective. While the affected ribosomal gene and mutation type play important roles in resulting phenotype, there are clearly many additional, yet unidentified, factors involved. Most of the family members in our study presented with steroid-responsive anemia in infancy and subtle congenital malformations, consistent with recent genotype-phenotype studies for *RPS19* and RPS vs*.* RPL*-*DBA (Arbiv et al., 2018; Iskander et al., 2021). Wide variations in phenotypic expression have been previously observed in families with both small deletions and missense mutations in *RPS19* (Willig et al., 1999; Ichimura et al., 2017; Da Costa et al., 2021). Missense mutations in *RPS19* have also been reported to be less penetrant than loss of function mutations (Ulirsch et al., 2018), which may help explain the two individuals in our study who were largely unaffected from a hematologic standpoint. However, those family members with significant anemia had varied clinical courses, which underscores the need for further studies to assess modifier genes, epigenetic and/or environmental factors which may result in differing responses to treatment.

DBA has recently been classified as a cancer predisposition syndrome of moderate penetrance, in which patients have a 4.8-fold higher relative risk of developing cancer (Lipton et al., 2021b). Colorectal cancer and osteogenic sarcoma are the most prevalent solid tumors in patients with DBA, and the Diamond Blackfan Anemia Registry (DBAR) has recently released early screening guidelines for CRC (Lipton et al., 2021b). At the present time, the median age for developing CRC in the setting of DBA is 41 years, though based on only nine patients (Lipton et al., 2021a). While the two individuals who developed CRC in our family do not meet the definition of early onset CRC (age of diagnosis <50 years), they still developed CRC prior to the median age in the general population of 66 years (Hofseth et al., 2020; Siegel et al., 2020). Although these two individuals may have other genetic or environmental risk factors that contributed to their cancers, their age of diagnosis highlights the importance of early cancer surveillance even in individuals with clinically mild DBA phenotypes. It is also important to note that it is atypical for DBA patients to tolerate chemotherapy as well as these two individuals did, and increased toxicity with prolonged cytopenias are more characteristic in the setting of DBA (Vlachos and Muir, 2010).

The mainstays of DBA treatment are aimed exclusively at ameliorating the defective erythropoiesis seen in DBA but these treatments are also associated with significant side effects. Long-term treatment with corticosteroids carries significant treatment morbidity including increased risk of hypertension, infection, diabetes mellitus, glaucoma, osteoporosis, and growth deficiency (Oray et al., 2016). Regular pRBC transfusions are used for patients who become corticosteroid resistant or intolerant, but also carry significant morbidity such as iron overload requiring chelation therapy (Vlachos et al., 2008; Vlachos and Muir, 2010). HSCT is curative, and patients less than 10 years of age were shown to have excellent chronic graft-versus-host disease-free survival for transplants performed in the past 2 decades (Strahm et al., 2020). There is, however, a high risk of transplant related mortality in older patients, as well as a risk of secondary malignancy after HSCT (Strahm et al., 2020; Diaz-de-Heredia et al., 2021).

Just as DBA is a heterogenous disease, individuals may also have varied choices in treatment, as evidenced by the individual in our family who prefers to live with severe anemia over the potential adverse effects of chronic corticosteroids or pRBC transfusions. Another family member enrolled in the Eltrombopag clinical trial in hopes that this medication targeted to increase RBC production will result in fewer side effects than his chronic transfusions. However, these treatment side effects could also potentially be improved if new therapeutics can selectively target the ribosomal defect itself.

L-leucine, a branch chain amino acid, is a potential emerging treatment for DBA, and other ribosomopathies, that upregulates ribosome biosynthesis via the mTOR signaling pathway and targets the underlying RP dysfunction (Payne et al., 2012; Xu et al., 2016; Orgebin et al., 2020). In a recent study, L-leucine treatment resulted in erythroid responses in a small number of transfusion-dependent patients with DBA, and improved weight gain and linear growth velocity in a larger number of patients (Vlachos et al., 2020). Trifluoperazine, a calmodulin inhibitor, is another emerging therapeutic treatment for DBA that has been shown to increase hemoglobin levels by reducing activation of p53 targets in both DBA animal models and patient-derived CD34^+^ cells (Taylor et al., 2020). Lastly, gene therapy is a promising therapeutic option, and a recent study demonstrated successful hematopoietic reconstitution using lentiviral vectors in *Rps19-*deficient DBA mouse models and in human CD34^+^ cord blood cells (Liu et al., 2022). Overall, these ribosome-targeted therapies are encouraging in that they may mitigate both the hypo-proliferative and hyper-proliferative (oncogenic) effects of ribosomal dysfunction.

Notably, our longitudinal study has provided 20 years of follow-up for this large family with DBA. This significant amount of time has given us the opportunity to observe the entire natural history of the disease including clinical phenotypes that have a long latency period (i.e., cancer), and ample time for patients to develop disease and/or treatment-related complications. However, this approach does have some limitations in that we require clinical updates through family questionnaires and clinical records from primary providers, which can lead to some incomplete information. For instance, most clinical details surrounding the death of patient 50-3 are unknown and thus it is unclear if her stroke may have been related to long term corticosteroid use. Additionally, patient 50-8 has not submitted clinical records since age 11 years, so it is unclear whether she has remained truly asymptomatic. Despite these inevitable challenges of natural history studies, they are still of utmost importance in advancing the understanding of rare diseases. In conclusion, DBA may be a different disease in every patient owing to the effects of genetic background and environment. DBA natural history studies are important to help elucidate yet unidentified factors that regulate phenotypic expression of a ribosomal genetic defect. Longitudinal follow-up is important to provide an understanding of factors that may potentiate treatment dependence or independence, and insight into cancer predisposition for those with clinically mild phenotypes. An understanding of factors influencing clinically milder phenotypes may provide insights into the pathophysiology of this ribosomopathy and ultimately lead to improved treatment options.

### Patient Perspective

Families tend to enroll in the IBMFS at the NCI to better understand the natural history of their disease and to have access to additional scientific and clinical expertise in DBA. Over the years, we have met the family in person for clinical evaluation and genetic testing and have also had countless emails and phone calls to discuss anything from DBA summer camp options to second opinions on cancer treatment.

## Data Availability

The datasets for this article are not publicly available due to concerns regarding participant/patient anonymity. Requests to access the datasets should be directed to the corresponding author.
